# Reasons to Keep “Case Reports” Alive

**DOI:** 10.21470/1678-9741-2020-0602

**Published:** 2020

**Authors:** Paulo Roberto B. Evora, Domingo M. Braile

**Affiliations:** 1 Editor-in-Chief Interim - BJCVS Faculdade de Medicina de Ribeirão Preto da Universidade de São Paulo (FMRP-USP), Ribeirão Preto, SP, Brazil.; 2 Editor-in-Chief - BJCVS Faculdade de Medicina de São José do Rio Preto (FAMERP), São José do Rio Preto, SP, Brazil and Universidade de Campinas (UNICAMP), Campinas, SP, Brazil.

Twenty years ago, many journals dropped case reports (CR) sections altogether, averaging only 15% of their published articles. It appears that two main forces had a role in the “Case Report” decline among medical publications: 1) the inability to use statistical methodology, and 2) the impact factor (IF). It is well known that a CR is usually cited no more than ten times within two years of being published, maybe having the lowest citation impact over all forms of medical literature. Based on these main limitations, the value of CR has been questioned, and many journals have abandoned it^[1,2]^.

**Fig. 1:**
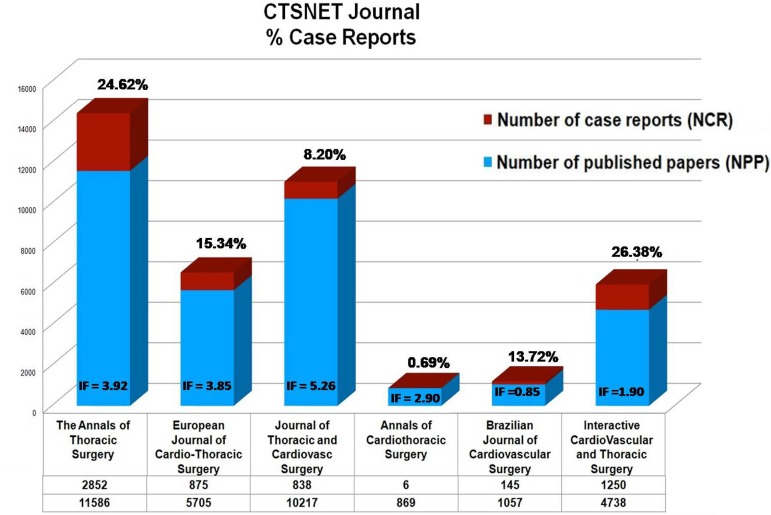
Case reports percentage and impact factor (IF) of some prestigious journals from The Cardiothoracic Surgery Network (CTSNET).

In a 10-year overview of CR published in The Cardiothoracic Surgery Network (CTSNET) journals, only three of them present IF higher than 3.0. The IF of the Journal of Thoracic and Cardiovascular Surgery is 5.262 and it shows 8.20% of CR, The Annals of Thoracic Surgery has IF of 3.919 and 24.62% of CR, and The European Journal of Cardio-Thoracic Surgery has IF of 3.847 and 15.34% of CR. This overview suggests that the CR restriction should be significant, but we cannot assume that the CR percentual was detrimental to the IF of the other three journals mentioned in Figure 1. By the way, the Brazilian Journal of Cardiovascular Surgery publishes 13,72% of CR and has IF of 0,85 (Figure 1).

Nowadays, we are experiencing a hard competition between publishers, more subliminally, competition in the pharmaceutical industry and the charging of publication fees, which are higher in journals with the highest IF. This detail alone is a factor of “exclusion” from low-economy countries. The high IF has its value. However, current conjuncture makes it clear that its obsessive pursuit can generate an invisible bias, which, in our opinion, can interfere with the democratic essence of science.

The academic and pharmaceutical worlds need to urgently review this tendency that has been supported by the referenced authors. “Case reports and case series may be the ‘lowest’ or the ‘weakest’ level of evidence, but they often remain the ‘first line of evidence’ and is where everything begins”, and maybe a starting point for hypothesis-testing research. Also, it is a potent argument in CR defense its long history of being an entry pathway for young physicians into the world of medical publication. Some ideas can be considered to keep CR “alive”: 1) CR database should be a great idea already adopted for many journals, and 2) CR would be presented as Educational, Image, or Multimedia presentations. These two suggestions are attractive options and may be stimulate^[3-5,9,10]^.

The desirable preservation of CR becomes a responsibility of the journal’s editorial staff under the guidance of their Editor-in-Chief. Maybe, the CR peer-review would be restricted to a specific group of Associate Editors, trying to get some criteria uniformity as suggested by the CARE Guidelines^[6,7]^.

“Last but not least, case reporting for medical education or medical research is great fun. Like much of medical reasoning, it has a detective-like quality. It brings a smile of recognition, or adequate understanding, to the faces of the presentation and audience. The temporary fall from favor of this classic type of medical literature may prove to have been the best remedy for its ultimate survival” (Vandenbrouke, 2001) ^[8]^.
